# Constant-distance mode SECM as a tool to visualize local electrocatalytic activity of oxygen reduction catalysts

**DOI:** 10.3762/bjnano.5.14

**Published:** 2014-02-07

**Authors:** Michaela Nebel, Thomas Erichsen, Wolfgang Schuhmann

**Affiliations:** 1 Lehrstuhl für Analytische Chemie; Ruhr-Universität Bochum, D-44780 Bochum; 2 Sensolytics GmbH, Universitätsstr 142, D-44799 Bochum

**Keywords:** electrocatalysis, oxygen reduction, recessed microelectrodes, redox-competition SECM, SECM, scanning electrochemical microscopy, shearforce-based constant-distance mode

## Abstract

Multidimensional shearforce-based constant-distance mode scanning electrochemical microscopy (4D SF/CD-SECM) was utilized for the investigation of the activity distribution of oxygen reduction catalysts. Carbon-supported Pt model catalyst powders have been immobilized in recessed microelectrodes and compared to a spot preparation technique. Microcavities serve as platform for the binder-free catalyst sample preparation exhibiting beneficial properties for constant-distance mode SECM imaging concerning modified surface area and catalyst loading. The integration of the redox competition mode of SECM into the detection scheme of the 4D SF/CD mode is demonstrated for specifically adapting high-resolution SECM experiments to powder-based catalyst preparations.

## Introduction

In scanning electrochemical microscopy (SECM) [1–2] an ultramicroelectrode, which is referred to as the SECM tip, is moved in close distance in a grid-wise manner over a sample surface. The resulting tip current is detected as a function of the tip position within the scanned grid, thus enabling visualization of the local electrochemical activity of the investigated sample surface caused by local variations of reaction rates. The tip current is, however, distance dependent and variations in the topography as well as in the local reactivity of the sample surface may both affect the tip response. Therefore, an unambiguous interpretation of the current response can only be realized by discrimination between the impact of topography and local reactivity by means of a constant-distance mode (cd-mode) positioning of the SECM tip in known and constant distance to the sample surface. The basis of cd-mode scanning is the control of the tip-to-sample separation by means of an additional electrochemistry-independent but distance-dependent analytical signal. Among other strategies for cd-mode imaging like the tip-position modulation mode (TPM) [3], AFM-SECM [4–5] and SECM-SICM [6–7], AC-SECM [8], the soft stylus probe [9–10] and the intermitted contact mode (IC-SECM) [11], the shearforce-based constant-distance mode [12–14] has been widely used and demonstrated its feasibility for high-resolution SECM. Furthermore, decoupling of the working distance of the electrochemical detection from the typically very short working distances required for cd-mode imaging was established recently proposing a 4D shearforce-based constant-distance mode (4D SF/CD-SECM) [15]. As a general strategy of tip movement it provides a tool to increase the analytical content of a single cd-mode SECM experiment and it is independent of the applied distance control mechanism. The concept of 4D CD-SECM was recently extended to intermittent contact-SECM [16].

Here, we describe the adaptation of the 4D SF/CD-SECM to the investigation of the activity of powdery oxygen reduction catalysts. In order to understand the properties of catalyst powders on a local scale, like, e.g., the distribution of activity within the catalyst material or initial processes of degradation, experiments have to be conceived which allow investigating the catalysts as close as possible to the conditions at which they are intended to be used. SECM is a promising tool in that respect. Typically, catalyst powders as samples for SECM investigations are immobilized as spots onto conductive plates such as, e.g., glassy carbon or conducting glasses. An example for such an investigation of catalyst libraries can be found in [17] and the local investigation of the activity of catalyst spots at elevated temperatures was recently described in [18].

In this contribution, we demonstrate the feasibility of 4D SF/CD-SECM to the topography-corrected investigation of the oxygen consumption profile of catalyst spots using commercially available model catalyst for the oxygen reduction reaction (ORR). Furthermore, recessed microelectrodes fabricated by etching inlayed Au disk microelectrodes are demonstrated as a flexible platform for immobilization of catalyst powders for cd-mode SECM experiments. The microcavities (also referred to as micropores) have already demonstrated their applicability for integral investigations of the activity of catalyst powders [19–26]. More recently recessed microelectrodes were already used for the visualization of powder catalyst activity using SECM [27–28]; however, cd-mode imaging and consequently topography-corrected activity determination was not described until now. Furthermore, the proposed 4D SF/CD mode provides the possibility to perform various complex electrochemical experiments at each grid point and in several known and constant distances to the contour of the sample surface. The combination of the transient redox competition mode (RC-SECM) [29–30] with the 4D SF/CD mode (by analogy named as 4D SF/CD-RC-SECM) for high-resolution SECM investigations of heterogeneous oxygen reduction catalysts is introduced as a strategy to further adapt the SECM detection scheme towards local visualization of ORR catalyst activity with high resolution.

## Results and Discussion

### Constant-distance mode imaging of catalyst spots

**Figure 1:**
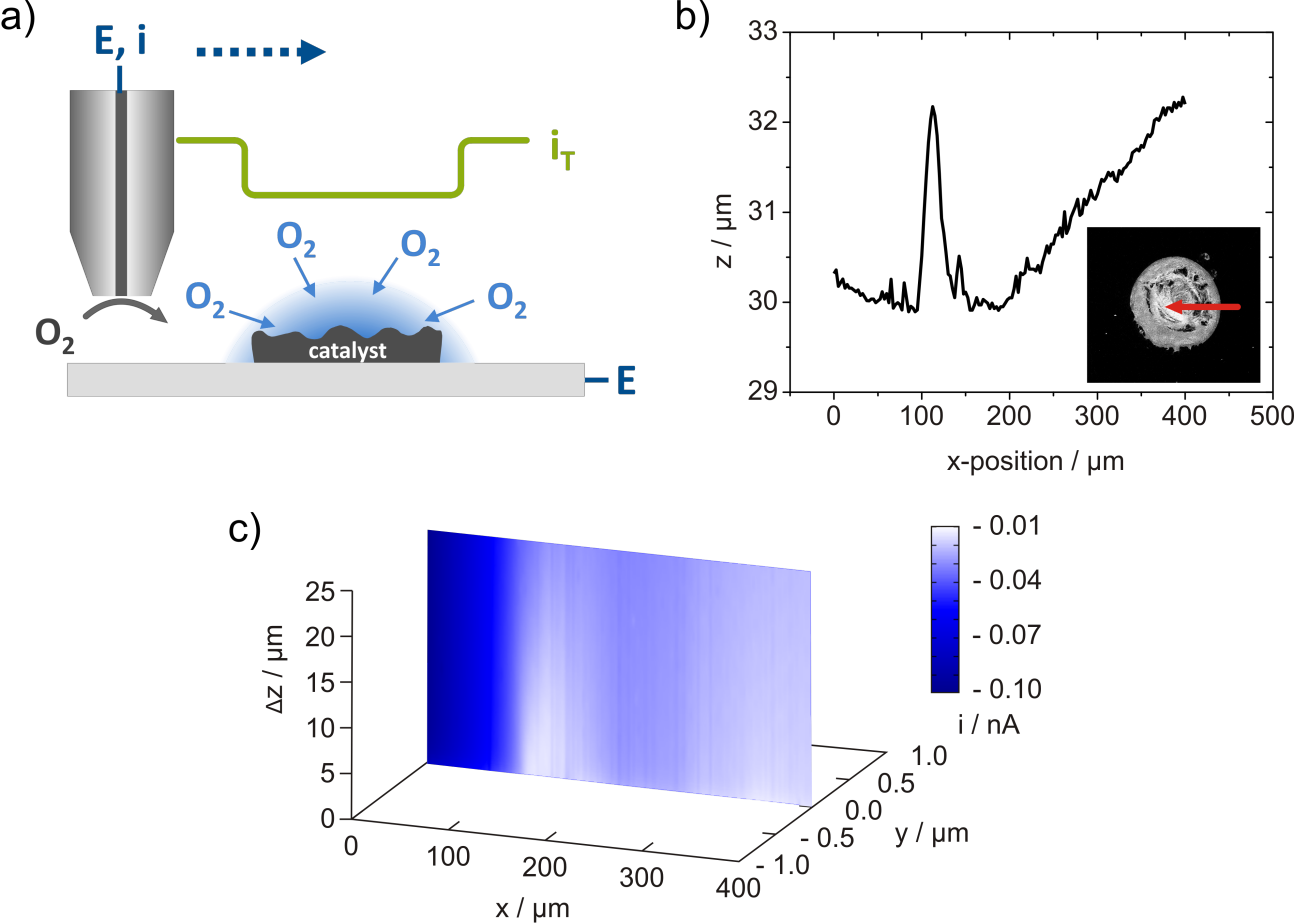
4D SF/CD-SECM for the investigation of the catalytic activity towards oxygen reduction. a) Scheme of the competition mode. b) Topographic linescan across a spot of Pt/C catalyst with 20 wt % Pt on a glassy carbon plate obtained in the shearforce-based cd-mode of SECM. The topography is displayed as the z-piezo position of the tip after reaching the predefined stop criterion of the shearforce interaction (point of closest approach). The insert shows an optical micrograph of the investigated spot and the arrow marks the scan position and direction. c) Oxygen consumption profile above the line displayed in b) visualized as a x,Δz,i-image, where Δz represents the distance of the current detection from the point of shearforce interaction. *E*_sample_ = −300 mV, *E*_tip_ = −600 mV, *d*_tip_ = 0.8 µm, *f*_osc_ = 356.7 kHz, stop criterion: 5% change of the lock-in signal in bulk.

The investigation of the activity of catalysts for ORR using SECM is commonly performed in a competition arrangement. A scheme of this variation of the generator/collector mode is shown in Figure 1a. The sample is polarized at a sufficiently low potential to reduce oxygen. Due to the catalyst reaction, oxygen is consumed and a gradual depletion of oxygen in front of the sample area occurs (consumption profile). Simultaneously, the tip is also polarized at a potential to reduce oxygen. Thus, sample and tip compete for the oxygen inside the gap between tip and sample which represents a leaking thin layer electrochemical cell. Due to the depletion of oxygen in the gap, a diffusion gradient is established leading to an in-diffusion of oxygen into the gap. Thus, depending on the tip-to-sample distance and the rates for oxygen reduction at tip and sample a stationary oxygen concentration is established within the gap. Hence, the tip current is modulated by the rate of oxygen reduction at the sample. The magnitude of current decrease at the tip in a competition experiment is visualizing the sample activity.

The topography image of a catalyst spot prepared from a commercially available ORR catalyst (Pt/C with 20 wt % Pt on Vulcan XC72 from ETEK) in a 4D SF/CD-SECM scan is shown in Figure 1b. The linescan started at a position above the glassy carbon plate and was performed towards the centre of the catalyst spot. Beside the formed outer ring with high catalyst loading due to a coffee drop effect [31–32], the topography of the catalyst spot was successfully visualized using shearforce-based positioning. The topographic features of the catalyst spot are also seen in the micrograph in the insert of Figure 1b. The oxygen consumption profile obtained during the same linescan is displayed in Figure 1c. The oxygen reduction current at the tip substantially decreases when the tip passes the border between glassy carbon plate and catalyst spot indicative for the ORR activity of the deposited powder catalyst. Variations of the tip current within the spot were detected showing a higher catalytic activity at positions with higher catalyst loading. Due to the cd-mode scanning, the current decrease above positions with higher catalyst thickness is independent from changes in the tip-to-sample distance and the observed tip response is therefore unambiguously allocated to the local catalyst activity for ORR. This experiment demonstrates the feasibility of the 4D SF/CD mode for studying the local activity of catalyst spots removing any impact from the sample topography. The accuracy of the shearforce-based tip positioning enables to follow the contour of the catalyst spot using comparatively stiff glass-insulated Pt tips in non-optical cd-mode. However, the visualization of the local catalytic activity of complete catalyst spots using SECM tips with sizes <5 µm is practicable only to a limited extent. The large size of the spots considerably increases the duration of cd-mode SECM experiments and electrolyte evaporation or electrode fouling may lead to unpredictable errors. The reduction of the time needed for a single SECM experiment using larger tip electrodes and/or increased distances between the grid points contradict the demand for high-resolution investigations. Using established spot preparation methods such as ink-jet dispensing it is possible to reduce the spot diameter to about 100 µm concomitantly depositing a substantially smaller catalyst loading leading to a decreased contrast in related SECM images. In order to overcome these drawbacks, an alternative sample preparation protocol based on the utilization of recessed microelectrodes as flexible platform for catalyst immobilization was applied for cd-mode SECM imaging.

### Microcavities as flexible platform for sample preparation in constant-distance mode SECM

**Figure 2:**
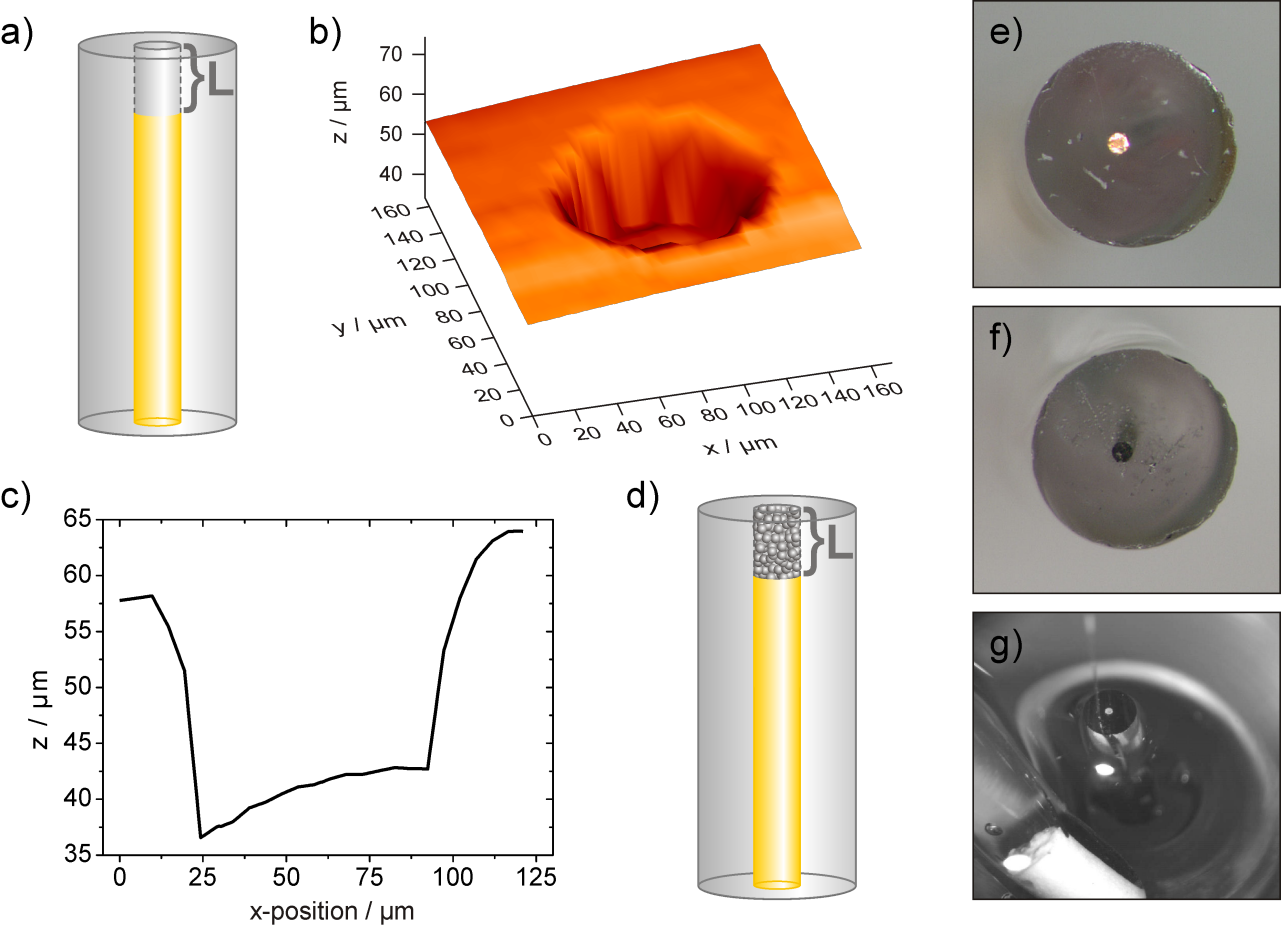
a) Scheme of a microcavity used as platform for catalyst immobilisation in cd-mode SECM investigations. b) Topography of the cavity obtained by means of shearforce-based cd-scanning and a corresponding linescan shown in c). d) Scheme of the microcavity electrode filled with catalyst powder. Optical micrographs of the cavity electrode before e) and after f) filling with catalyst powder. Residues of the black Pt/C catalyst are visible at the glass insulation and are removed before the filled cavity is inserted in the SECM measuring cell. g) Micrograph of the setup for 4D SF/CD-SECM experiments with the tip positioned next to the microcavity. The glass body of the used reference electrode is visible in the front.

The requirement of a small catalyst-modified area with simultaneously high catalyst loadings are met by the application of recessed microelectrodes whose cavities are filled with the desired catalyst powder. The bottom of the microcavity forms the electrical contact and the thickness of the investigated catalyst material is determined by the depth *L* of the cavity (see Figure 2a). This sample preparation is fast, enables an easy exchange of catalyst powder, is applicable for quantitative studies and the modified surface area is determined by the opening of the cavity [25–26]. The fabrication of nanometre-sized recessed electrodes have already been reported [33–34] and a further miniaturization of the modified surface area is therefore possible. Furthermore, immobilization of the catalyst powder within the cavity of the recessed microelectrode allows for avoiding any binder additive such as, e.g., Nafion that is commonly used for rotating disk electrode studies or spot preparation [23]. Since the influence of Nafion on the catalytic activity is still not fully understood [35–37], the investigation of the catalyst material in absence of any binder is an additional advantage of this sample preparation method. In order to generate a microcavity, polished disk-shaped gold electrodes with a diameter of 100 µm were electrochemically etched in HCl using 10 potentiodynamic cycles. The depth of the formed cavity was investigated by shearforce-based cd-mode scanning and a complete topography image and a corresponding topographic linescan across the centre of the cavity are displayed in Figure 2b and Figure 2c, respectively.

**Figure 3:**
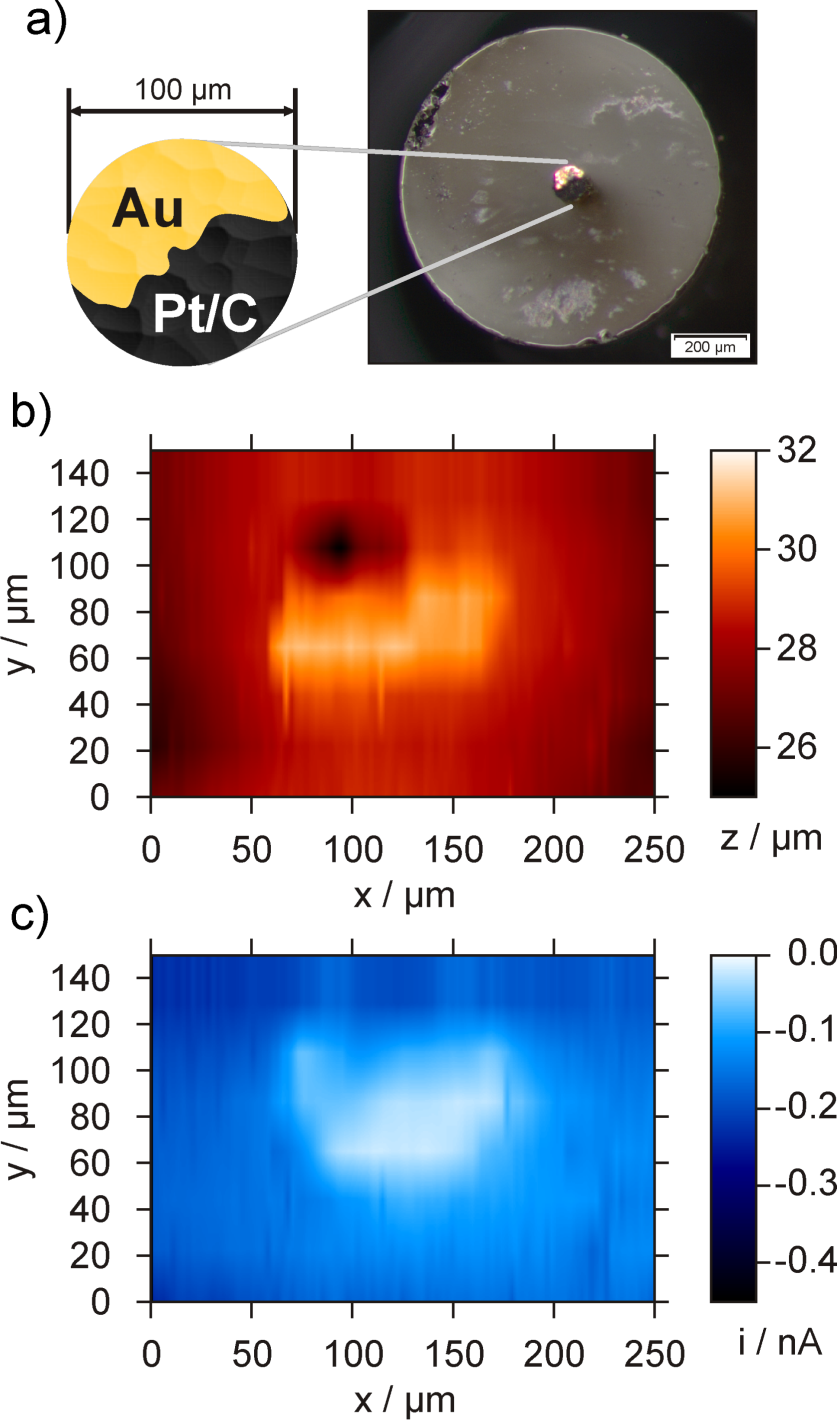
4D SF/CD-SECM experiment at a partly filled microcavity. a) Optical micrograph with a scheme of the catalyst filling, b) topography and c) current image at the point of closest approach. *E*_sample_ = −300 mV, *E*_tip_ = −600 mV, *d*_tip_ = 1.6 µm, *f*_osc_ = 299.4 kHz, stop criterion: 15%.

The depth *L* was determined to be about 19 µm with an inhomogenous contour of the bottom of the microcavity. Obviously, the material removal at the edges of the cavity was more efficient than at the centre leading to a protruding structure of the cavity bottom. Similar results were previously shown for chemically etched Pt disk electrodes [25]. However, the non-flat cavity bottom does not impede with the function as a conductive contact for the catalyst powder filled into the cavity. The depth of about 19 µm allows high catalyst loadings making any impact of bottom irregularities on the activity determination being negligible. The microcavities were further used for catalyst immobilization and manually filled with the catalyst powder (Figure 2d). The result of the filling procedure was examined by means of optical microscopy (Figure 2e and Figure 2f). Subsequently, the prepared catalyst-filled microcavity electrode was integrated into a specifically designed measuring cell for cd-mode SECM experiments (Figure 2g). As a matter of fact, it is important to elucidate that the Au bottom of the recessed microelectrode does not have a major impact on the measured catalytic activity for ORR. A partly filled microcavity (Figure 3a) was analysed by means of 4D SF/CD-SECM in the competition arrangement. Although the tilt between the scanning plane and the surface of the microcavity electrode was compensated by the distance control it was visible in the topography image displaying the z-position of the tip when the stop criterion of the shearforce signal was obtained. In order to enhance the contrast, the tilt was subtracted and the visualization of the resulting sample topography is shown in Figure 3b. The lateral inhomogeneous filling of the microcavity with the catalyst powder is clearly visible. With respect to the surrounding glass plane the catalyst filling is elevated by about 1 to 2 µm. Additionally, the unfilled part of the cavity can be distinguished, however, due to the size of the glass sheath of the tip, an edge effect is obtained allowing the tip only to move into the cavity at distances from the edge being larger than half of the overall tip diameter. The tip current at closest tip approach is displayed in Figure 3c showing a decrease of the tip current exclusively above areas filled with catalyst material. The current detection of the 4D SF/CD mode is sensitive enough to visualize the fine structure of the filling and even the thin catalyst layer at the left side of the cavity was distinguished as a location of higher catalytic activity. No interference of the free Au bottom was detected at an applied sample potential of -300 mV.

### 4D constant-distance mode RC-SECM (4D SF/CD-RC-SECM)

**Figure 4:**
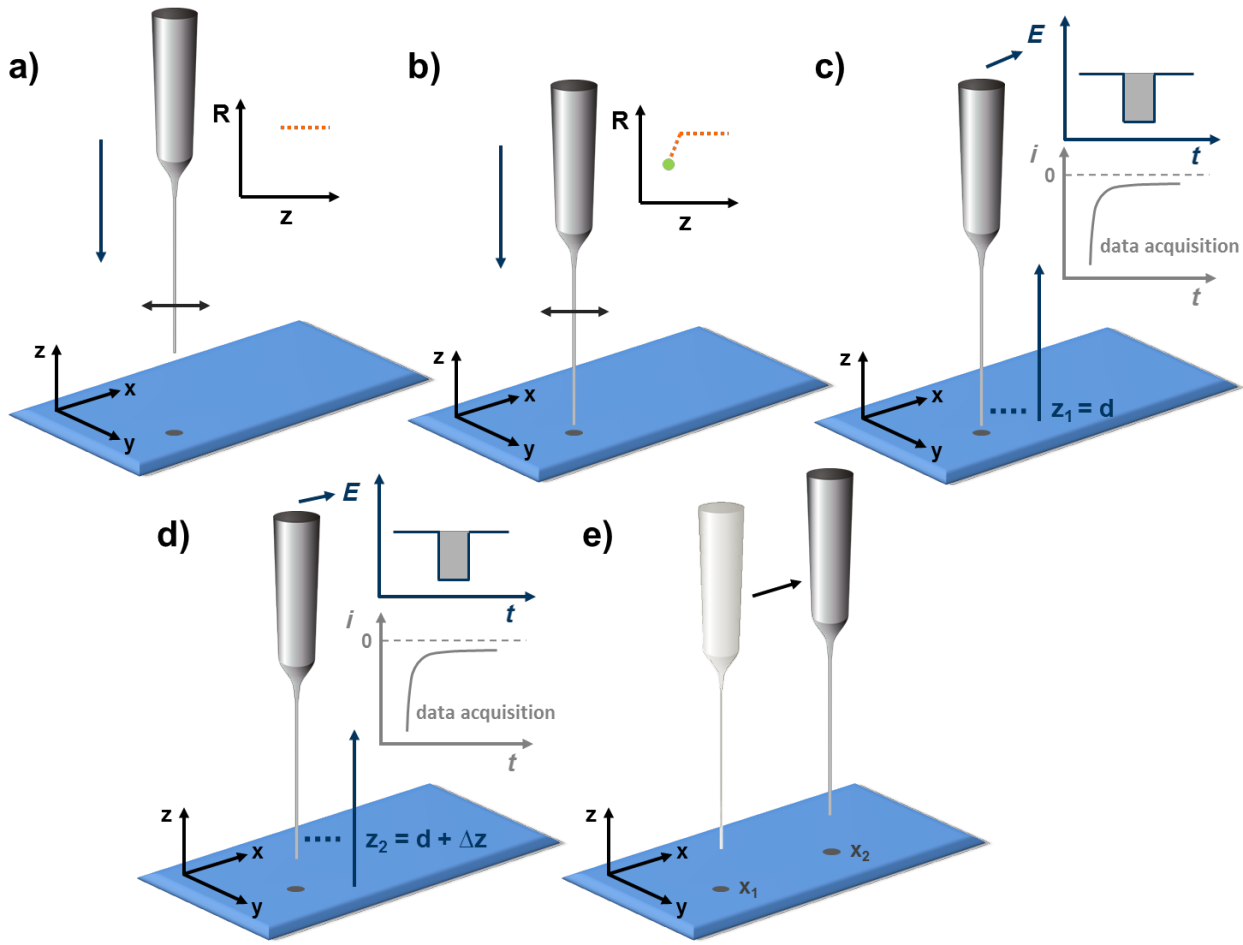
Concept of the 4D SF/CD-RC-SECM. Similar to the 4D SF/CD mode the tip is positioned within the shearforce-interaction regime by means of a z-approach curve (a and b). Data acquisition is performed in subsequent retraction steps (c and d) set out from the point of closest approach. To additionally implement the redox competition mode, a variable potential pulse profile is performed at each tip-to-sample distance. During a competition pulse tip and sample compete for the oxygen inside the gap and a time-dependent current decay curve is recorded at the tip enabling a time-resolved analysis of the oxygen concentration within the gap. After completion of the retraction the tip is moved to the next grid point (e) and the procedure is repeated starting with the shearforce-based positioning (a).

The redox competition mode of scanning electrochemical microscopy (RC-SECM) is a specially designed SECM detection mode for the investigation of oxygen reduction catalysts [29–30]. The current measurement is performed at the tip and a further increase of the lateral resolution by utilization of smaller electrodes is therefore possible [29]. In order to enable a precise positioning of small electrodes also for the RC mode, the detection scheme was integrated in the 4D SF/CD-SECM. This combination leads to the concept of 4D SF/CD-RC-SECM as illustrated in Figure 4. As a variation of the 4D shearforce-based constant-distance mode the tip is vibrated at its own resonance frequency and approached towards the sample surface under shearforce control until the predefined stop criterion is reached (Figure 4a). After precise positioning of the tip within the shearforce interaction regime, the resulting z-position is stored to visualize the sample topography (Figure 4b). The concept of 4D SF/CD-SECM provides the possibility to perform any anticipated electrochemical experiment at each grid point and each tip-to-sample separation. Integration of the transient redox competition mode of SECM is performed by implementing a potential pulse profile that is applied at the tip and the time-resolved detection of the current response is performed (Figure 4c and 4d). After completion of the stepwise retraction the tip is moved to the next grid point (Figure 4e) and the procedure of shearforce-based positioning and retraction with simultaneous data acquisition is repeated. By using the constant-distance mode RC-SECM, local chronoamperograms are recorded at different but constant tip-to-sample separations at each point of the scanned grid. This enables the determination of the complete time-depended oxygen consumption profile in constant tip-to-sample separations far outside the restricted range of the shearforce interaction. In accordance with the previously proposed terminology of multidimensional SECM and the transient character of the 4D SF/CD-RC-SECM, this mode is a 5D measuring technique (*i* vs *t* vs x-, y-, z-position). The representation of the multidimensional data set is optionally realized by horizontally sliced current images in different tip-to-sample distances at discrete points of time. Alternatively, a set of vertical oxygen consumption profiles can be extracted.

**Figure 5:**
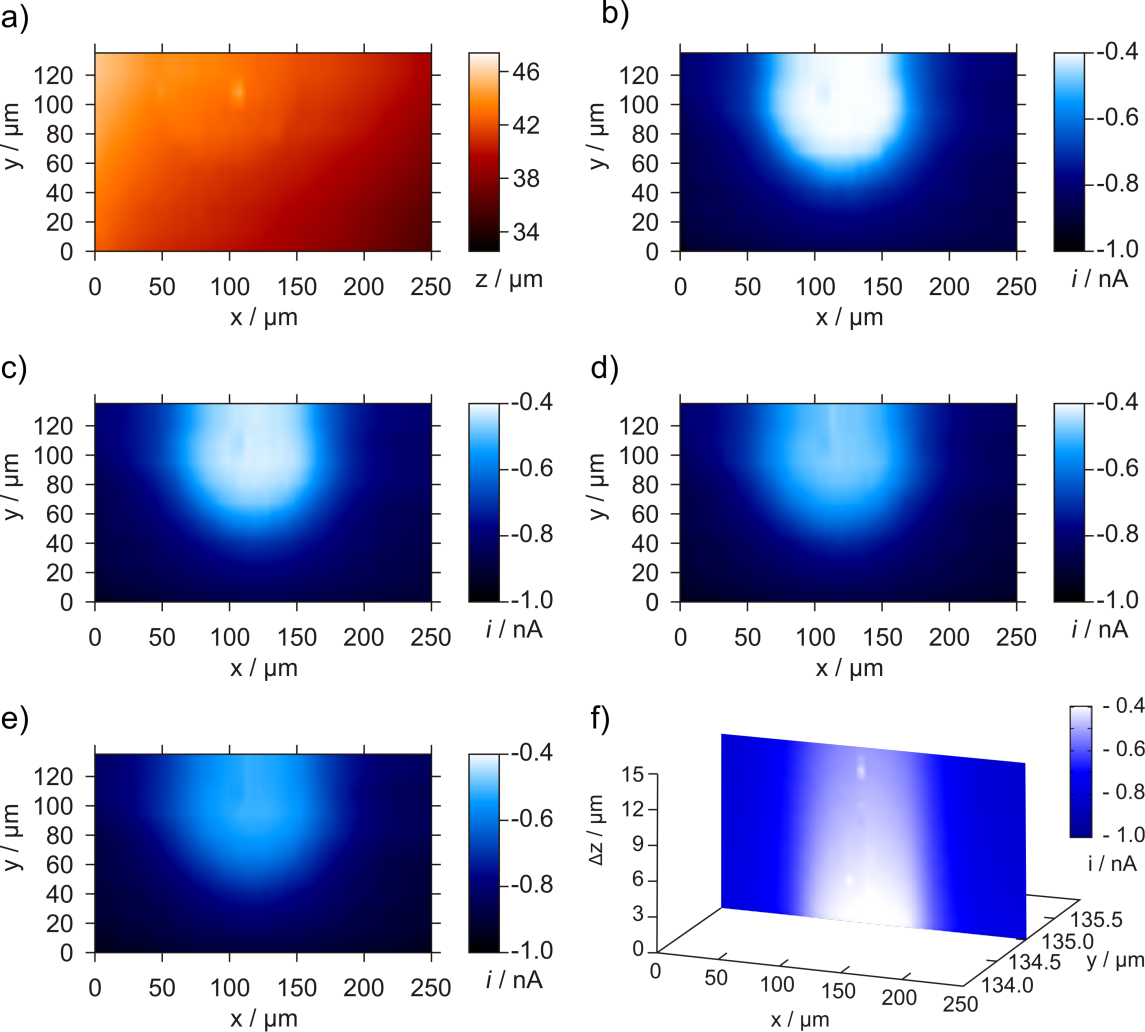
4D SF/CD-RC-SECM experiment at a 100 µm diameter Pt disk electrode as model sample for an ORR catalyst. a) Topography image, b)–e) horizontal current image at the point of shearforce contact and in increments of 5 µm after tip retraction. f) Oxygen consumption profile (vertically-sliced current image). *E*_sample_ = −600 mV, *E*_tip,base_ = 0 mV, *E*_tip,pulse1_ = 0 mV, *t*_pulse1_ = 0.5 s, *E*_tip,pulse2_ = −600 mV, *t*_pulse2_ = 0.5 s, pictures displayed at *t* = 0.25 s, *d*_tip_ = 3.4 µm, *f*_osc_ = 318.2 kHz, stop criterion: 5%.

Figure 5 shows the application of the 4D SF/CD-RC mode to a polished Pt disk electrode as model sample for an ORR catalyst.

**Figure 6:**
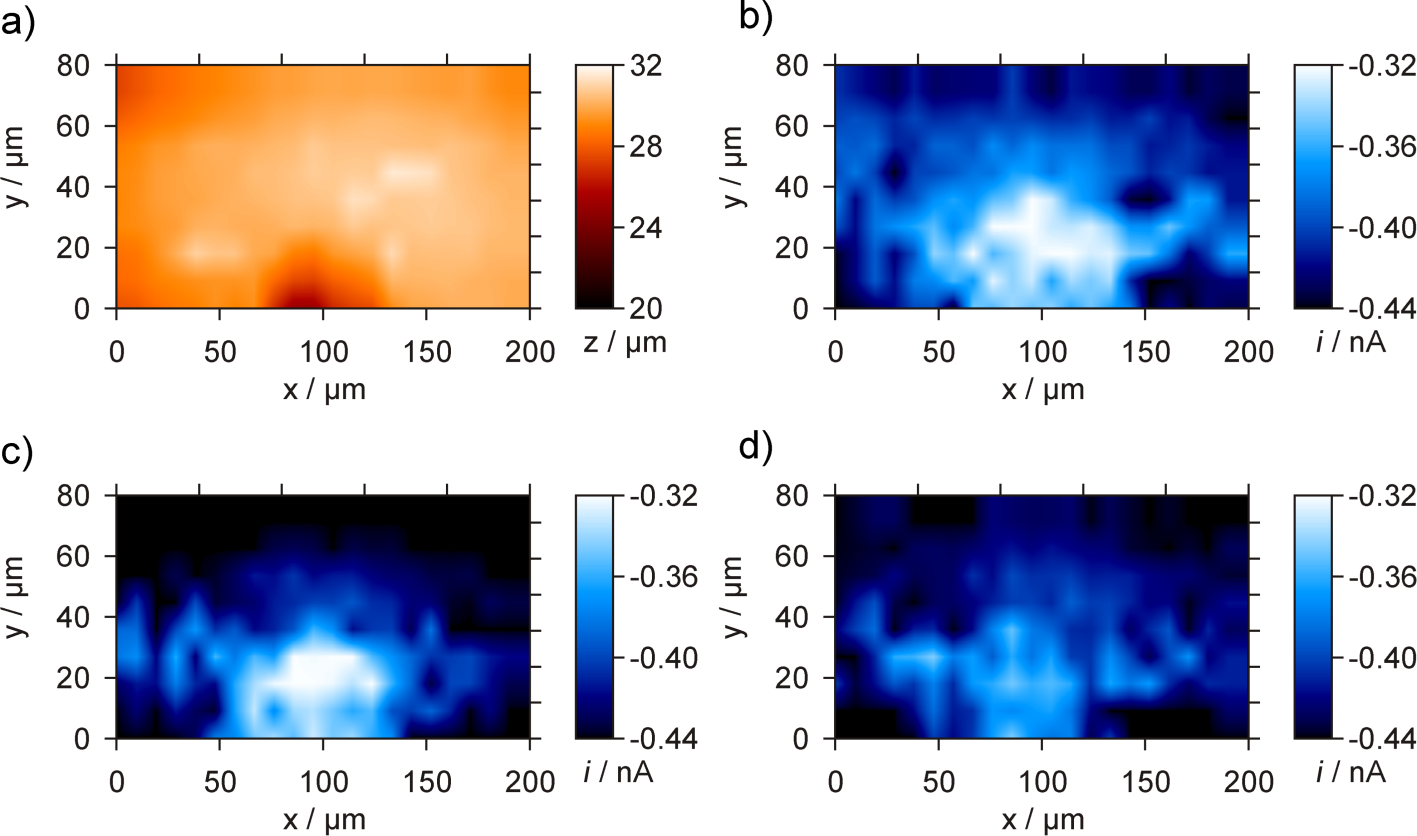
4D SF/CD-RC-SECM experiment at a microcavity filled with a Pt/C model catalyst. Topography image a) and current images at b) the point of shearforce-contact *d*, c) *d* + 4 µm, and d) *d* + 20 µm. *E*_sample_ = −300 mV, *E*_tip,base_ = 500 mV, *E*_tip,pulse1_ = 500 mV, *t*_pulse1_ = 0.5 s, *E*_tip,pulse2_ = −600 mV, *t*_pulse2_ = 0.5 s, images displayed at *t* = 0.25 s, *d*_tip_ = 1.9 µm, *f*_osc_ = 366.7 kHz, stop criterion: 5%.

A tilt of the surface is visible in the topography image (Figure 5a) and the Pt surface is elevated by about 200 nm above the glass insulation. The area above the active Pt disk is clearly distinguishable as a location with increased oxygen consumption (Figure 5b–e). The contrast decreases with larger working distances representing the diminution of the vertical oxygen consumption profile as displayed in Figure 5f. This result shows the feasibility of the proposed 4D SF/CD-RC-SECM for the visualization of the local catalyst activity for ORR. In order to proof the applicability of the 4D SF/CD-RC-SECM for the evaluation of the catalytic activity of powder catalysts, a Pt/C filled microcavity was investigated (Figure 6).

The filled cavity is visible as a maximal 3 µm recessed area in the topography image displayed in Figure 6a. A decreased oxygen concentration was successfully detected above the filled cavity and the current images at increasing tip-to-sample separations (Figure 6c and d) show the expansion of the oxygen consumption profile into the electrolyte solution.

Apart from the local catalytic activity, the contrast of a RC-SECM experiment depends on several parameters like, e.g., the RG value and the size of the tip, the tip-to-sample separation, the applied pulse profile and the time of data acquisition [29]. Due to this complex multiparameter system, the experimental conditions for the best data acquisition varies for each measurement. By means of the 4D SF/CD-RC-SECM a technique is accessible that enables a time-dependent data acquisition at various constant working distances. As a result, the conditions for the best contrast of a SECM experiment are chosen after the SECM investigation is completed without the demand of knowing the exact contribution of each parameter beforehand. Moreover, we recently demonstrated that the reaction at a continuously polarized tip may actively influence the detection of the local oxygen concentration inside the gap between tip and sample [38–39]. As a consequence, the oxygen detection is superimposed by effects caused by the tip reaction and the tip is not acting as a passive spectator. Using the flexible potential pulse profile of the 4D SF/CD-RC mode the tip can be polarized to the oxygen reduction potential for only a very short time. Thus, the diffusion field in front of the electrode is restricted and an influence of the tip reaction on the imaging result is minimized.

## Conclusion

The application of the 4D shearforce-based constant-distance mode for the investigation of the activity of ORR catalyst powders enables the visualization of the activity distribution within a catalyst spot avoiding any convolution due to topography effects. Catalyst-filled microcavities were successfully used as platform for the immobilization of catalyst powders. No influence of the underlying gold support on the determined local catalyst activity has been detected. Additionally, integration of RC-SECM in the detection scheme of the 4D SF/CD mode was successfully applied for the investigation of catalyst filled microcavities.

Catalysts for industrial applications are typically operated at elevated temperatures. Due to the availability of a temperature-controlled SECM setup [18] and the recently demonstrated capability of the tip-to-sample distance determination via shearforce-based approach curves in front of a heated surface [40], future work aims towards the high-resolution detection of catalytic activity at elevated temperatures.

## Experimental

### Chemicals and materials

All chemicals were used as received without further purification and all aqueous solutions were prepared using ultrapure water (Siemens Water Technologies, Barsbüttel, Germany). The electrochemical characterization of the SECM tips, microelectrodes, and recessed microelectrodes as sample surfaces were performed by means of cyclic voltammetry in an electrolyte containing 5 mM [Ru(NH_3_)_6_]Cl_3_ (ABCR, Karlsruhe, Germany) and 100 mM KCl (Riedel-de Haën, Seelze, Germany) as background electrolyte. As a model catalyst a commercial nanoporous ORR catalyst from ETEK (Natick, USA) with 20 wt % Pt on Vulcan XC72 was used. An air saturated 0.1 M sodium phosphate buffer (pH 7, VWR International, Darmstadt, Germany) was used for all investigations of the catalytic activity towards ORR.

Platinum wires with diameters of 25 µm, 100 µm and 250 µm and a gold wire with a diameter of 100 µm were from Goodfellow (Bad Nauheim, Germany). Borosilicate glass capillaries (length 100 mm, outside Ø 1.5 mm, inside Ø 0.75 mm) were purchased from Hilgenberg (Malsfeld, Germany) while quartz glass capillaries (length 95 mm, outside Ø 0.9 mm, inside Ø 0.3 mm) were from Quarzschmelze Illmenau (Illmenau, Germany).

### SECM instrumentation and tip preparation

A specifically designed SECM setup was used for all experiments and the main components have been described previously [15]. The SECM tip was stationary and the electrochemical measuring cell with the sample at the bottom movable by a stage of three joined stepper motors (SPI Robot Systems, Oppenheim, Germany). The nominal resolution in x-, y-, and z-direction was 10 nm per microstep. Additionally, a three axis piezo-positioning system (Nanocube P-611.3S, Physik Instrumente, Waldbronn, Germany) with strain gauge position sensors was used. The total travel length was 100 µm in each direction with a minimum step width of 1 nm and a reproducibility <10 nm.

The 4D shearforce-based constant-distance mode [15] with non-optical shearforce detection was used for all SECM measurements. A piezoelectric detection system (Sensolytics, Bochum, Germany) that consists of two piezoelectric plates each of them glued onto a brass holder was used for the detection of the shearforce interaction. For the piezo-piezo detection these piezo elements have been directly attached to the electrode body as reported in [13,41]. The needle shaped electrode is set into vibration at its own resonance frequency (*f*_osc_) by means of the first attached piezo element. Changes of the vibration magnitude and phase shift are detected by the second piezo element that is closely positioned at the very end of the non-tapered electrode body. Excitation and low-noise determination of the tip vibration were realized by a lock-in amplifier (model 7280, Signal Recovery, Wokingham, UK). Electrode potentials and data acquisition were controlled by a bipotentiostat model PG 100 (Jaissle Elektronik, Waiblingen, Germany) in combination with a 16 bit AD/DA board (PCI-2517, Plug-In Electronics, Eichenau, Germany). In order to minimize electrostatic noise, the whole system was placed in a Faraday cage. The in-house written software for controlling the SECM setup was programmed in Visual Basic 6.0 (Microsoft, Unterschleissheim, Germany). The tip movement of the 4D shearforce-based constant-distance mode includes a shearforce-based approach curve until a predefined vibration change (stop criterion, typically in the range of 5–15%) is reached. Subsequently, a stepwise retraction of the tip with simultaneous data acquisition at each pre-defined tip-to-sample separation is performed. Diffusion profiles exceed far the geometric dimensions of the investigated surface structure. The collection of electrochemical data in z-direction requires therefore comparable large distances towards the sample surface. After lateral displacement, the tip is approached until the shearforce interaction range (usually observed at distances <300 nm) is reached again. In order to decrease the timescale of the tip approach, the procedure of the 4D SF/CD-SECM was supplemented with a predefined distance that is approached with a fast speed (typically 10 µm/s). Only the last few microns are afterwards approached at slow speed (e.g., 0.5 µm/s) that is required for the accurate shearforce-based positioning.

Vibratory needle shaped SECM tips were fabricated by means of a laser puller P-2000 from Sutter Instruments (Novato, USA) following an earlier published procedure [42]. Parameters for the sealing step were: Heat 810, Filament 5, Velocity 100, Delay 120, Pull 1 (repetition of 8–11 cycles, 20 s on and 40 s off). For the pulling step Heat 870, Filament 5, Velocity 130, Delay 150, Pull 220 was used. The platinum of the fabricated SECM tips was exposed by cutting the very end of the pulled Pt/capillary assembly with a blade under microscopic control (BX41, Olympus, Hamburg, Germany). For polishing a machine like in [42] was used. The electrodes were characterized by means of optical inspection under the microscope and by cyclic voltammetry with [Ru(NH_3_)_6_]^3+^ as redox mediator. The size of the disk shaped electrodes was calculated using the diffusion limited steady state current and *i* = 4*nFDcr* (*n*: number of electrons transferred per molecule, *F*: Faraday constant, *D*: diffusion coefficient, *c*: bulk concentration of the electroactive species and *r*: radius of the active electrode surface). A diffusion coefficient of 9.1 × 10^−6^ cm^2^s^−1^ for [Ru(NH_3_)_6_]^3+^ in KCl [43] was used. In all experiments a coiled Pt-wire (Ø 250 µm) counter electrode and a home build miniaturized Ag/AgCl (3 M KCl) reference electrode was used.

### Sample Preparation

#### Catalyst spots

Spots of ORR catalyst powders were prepared by using a catalyst suspension applied by means of a piezoceramic spotter (model SciFA DW with tip PDC 80 from Scienion; Dortmund, Germany) onto polished (0.3 µm alumina paste) glassy carbon plates (Sigradur G, 1 mm thickness, HTW, Thierhaupten, Germany). 1 mL of the catalyst suspension consisted of 2.5 mg catalyst powder in a solution of 49.5% ethanol, 49.5% water and 1% Nafion (solution of 5 wt % Nafion from Sigma-Aldrich, Steinheim, Germany). The spot was prepared by 3200 droplets dispensing 45 pL of catalyst suspension per droplet.

#### Microelectrodes and microcavities

All SECM investigations of microelectrodes and microcavities as SECM sample were performed with a specifically designed measuring cell that enables an upstanding positioning of the electrode relative to the SECM tip (see also Figure 2g). All electrodes used as sample surface in SECM experiments had a total length of 2.5 cm. They were screwed upright inside the bottom of the measuring cell via a fitting and a corresponding O-ring. A polished Pt disk electrode with a diameter of 100 µm embedded in a large glass sheath with an overall outer diameter of 1.5 mm was used as a model sample for 4D SF/CD-RC-SECM experiments. Microcavities as platforms for catalyst immobilization were prepared via electrochemical etching of Au microelectrodes with diameters of 100 µm. After polishing of the initial Au disc electrode and cleaning for 15 min in distilled water in an ultrasonic bath electrochemical cleaning by means of potentiodynamic cycling (potential range: 0 mV to 1700 mV, scan rate: 200 mV s^−1^, 20 cycles) in 0.5 M sulfuric acid and subsequent electrochemical characterisation of the cleaned disk electrode was performed. Electrochemical etching was performed in 6 M HCl by cyclic voltammetry (potential range: 100 mV to 1300 mV, scan rate: 500 mV s^−1^, 10 cycles). The chloride ions act as a complexing agent and at a potential of about 1100 mV etching of gold is performed [44]. After completion of the etching remaining impurities were removed by an ultrasound washing step in water and electrochemical cleaning in H_2_SO_4_. The recessed electrode was characterised by means of CV in 5 mM [Ru(NH_3_)_6_]Cl_3_ and 100 mM KCl. The formed microcavity was filled with the catalyst powder by pressing the etched electrode in a small amount of catalyst material on a glass slide. Circular movements of the electrode on the glass plate lead to a homogeneous filling of the cavity. Excess catalyst material was removed with a tissue under microscopy control until the catalyst filling was lined up precisely with the surrounding glass sheath.
